# Inhibitors targeting Bruton’s tyrosine kinase in cancers: drug development advances

**DOI:** 10.1038/s41375-020-01072-6

**Published:** 2020-10-29

**Authors:** Tingyu Wen, Jinsong Wang, Yuankai Shi, Haili Qian, Peng Liu

**Affiliations:** 1 Department of Medical Oncology, National Cancer Center/National Clinical Research Center for Cancer/ Cancer Hospital, Chinese Academy of Medical Sciences and Peking Union Medical College, 100021 Beijing, China; 2 State Key Laboratory of Molecular Oncology, National Cancer Center/National Clinical Research Center for Cancer/Cancer Hospital, Chinese Academy of Medical Sciences and Peking Union Medical College, 100021 Beijing, China

**Keywords:** Drug development, Molecularly targeted therapy

## Abstract

Bruton’s tyrosine kinase (BTK) inhibitor is a promising novel agent that has potential efficiency in B-cell malignancies. It took approximately 20 years from target discovery to new drug approval. The first-in-class drug ibrutinib creates possibilities for an era of chemotherapy-free management of B-cell malignancies, and it is so popular that gross sales have rapidly grown to more than 230 billion dollars in just 6 years, with annual sales exceeding 80 billion dollars; it also became one of the five top-selling medicines in the world. Numerous clinical trials of BTK inhibitors in cancers were initiated in the last decade, and ~73 trials were intensively announced or updated with extended follow-up data in the most recent 3 years. In this review, we summarized the significant milestones in the preclinical discovery and clinical development of BTK inhibitors to better understand the clinical and commercial potential as well as the directions being taken. Furthermore, it also contributes impactful lessons regarding the discovery and development of other novel therapies.

## Introduction

B-cell malignancies include non-Hodgkin lymphomas (NHLs) and chronic lymphocytic leukaemia. Approximately 93% of NHLs are derived from B cells, and B-cell malignancies comprise the most common haematologic malignancy with an estimated 98,280 new cases and 24,000 deaths in the US in 2020 [1, 2]. The most common subtypes include chronic lymphocytic leukaemia/small lymphocytic lymphoma (CLL/SLL), diffuse large B-cell lymphoma (DLBCL), follicular lymphoma (FL), multiple myeloma (MM), marginal zone lymphoma (MZL), mantle cell lymphoma (MCL) and Waldenström’s macroglobulinemia (WM). NHL is the third indication (9%) followed by solid tumours (23%) and non-small cell lung cancer (17%) for new drug clinical trials in mainland China from 2009 to 2018 [3]. Notably, B-cell malignancies had one of the highest clinical trial transition success rates among cancers. The successful probability rates from the phase I trial to FDA approval in NHL, MM, and CLL were ~8.5%, 9.7%, and 7.3%, respectively, compared with 5.7% in total solid tumours [4]. As such, tremendous progress regarding the therapy of B-lymphoid malignancies has been achieved and dramatically improved patient outcomes, especially for frail elderly patients in the past two decades.

**Fig. 1:**
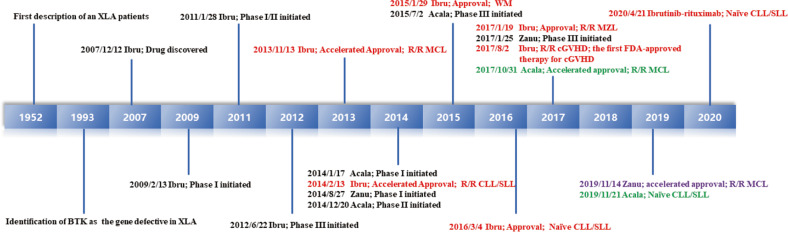
Key milestones in the target discovery and clinical development of BTK inhibitors, with United States’ FDA approved indications. The BTK gene is first identified in 1993, and the BTK inhibitor ibrutinib is designed for clinical trials in 2009. It takes approximately 20 years from target discovery to new drug approval. As follows, the second-generation BTK inhibitors of acalabrutinib and zanubrutinib were launched in 2014, and approved by FDA in 2017 and 2019, respectively.

Inhibitors targeting Bruton’s tyrosine kinase (BTK) are novel agents for NHL, and it has created possibilities for an era of chemotherapy-free management of B-cell malignancies. Since the structure and function of BTK was well defined in 1993 [5], there were numerous investigations from industry and academia to develop BTK inhibitors as antitumour agents or beyond (Fig. 1). Ibrutinib was the first effective and selective BTK inhibitor approved by the FDA as a breakthrough therapy in 2013. Its approval has had epoch-making significance. Because toxic chemotherapy is the main option for CLL/SLL before its initiation, it brings the concept of chemotherapy-free management to B-cell malignancies. It was so popular that the gross sales rapidly grew to more than 230 billion dollars in just 6 years, with annual sales exceeding 80 billion dollars, and it became one of the five top-selling medicines in the world. Subsequently, the second-generation BTK inhibitors acalabrutinib and zanubrutinib, which tried to reduce off-target effects, were approved in 2017 and 2019, respectively. Over the past decade, numerous preclinical and clinical studies are evaluating the efficacy of BTK inhibitors as single agents or in combination with other standard chemotherapy, immunotherapy, or targeted agents in various cancers to broaden indications and expand markets. Given that it often takes approximately 3–6 years from developing an investigational new drug to yielding new drug applications, the results were intensively announced or updated in the most recent 3 years. Thus, we systematically reviewed available BTK inhibitor preclinical and clinical data to provide insight on changes in the drug development process of BTK inhibitors and identify unmet clinical needs.

### BTK is a critical molecule that interconnects BCR signalling, Toll-like receptor (TLR) signalling, and chemokine receptor signalling

**Fig. 2:**
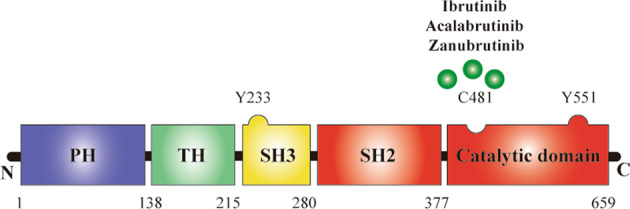
Structure of BTK. BTK is consisted by 659 amino acids, contains five domains, from N-terminal to C-terminal, domains are listed as the Pleckstrin homology (PH) domain, proline-rich TEC homology (TH) domain, SRC homology (SH) domain SH3, SH2, and the catalytic domain. Now, three approved BTK inhibitors are mainly targeted in the catalytic domain of the BTK.

In 1993, Vetrie identified that the primary immunodeficiency disease X-linked agammaglobulinemia (XLA) is caused by gene mutations and named this gene in Xq22.1 BTK. BTK consists of 659 amino acids and five domains: the pleckstrin homology (PH) domain, the proline-rich TEC homology (TH) domain, the SRC homology (SH) domains SH3 and SH2, and the catalytic domain from the N-terminus to the C-terminus (Fig. 2). The PH domain can bind to phosphatidylinositol lipids, such as PIP3, and recruit proteins to the cell membrane. The TH domain contains a zinc-finger motif that is important for optimal activity and stability of the protein. The SH domains are involved in protein–protein interactions and bind to phosphorylated tyrosinase and proline-rich regions. The catalytic kinase domain Y551 site can be phosphorylated either by LYN Proto-Oncogene (LYN) or Spleen tyrosine kinase (SYK) and result in autophosphorylation of the SH3 domain Y233 position.

**Fig. 3:**
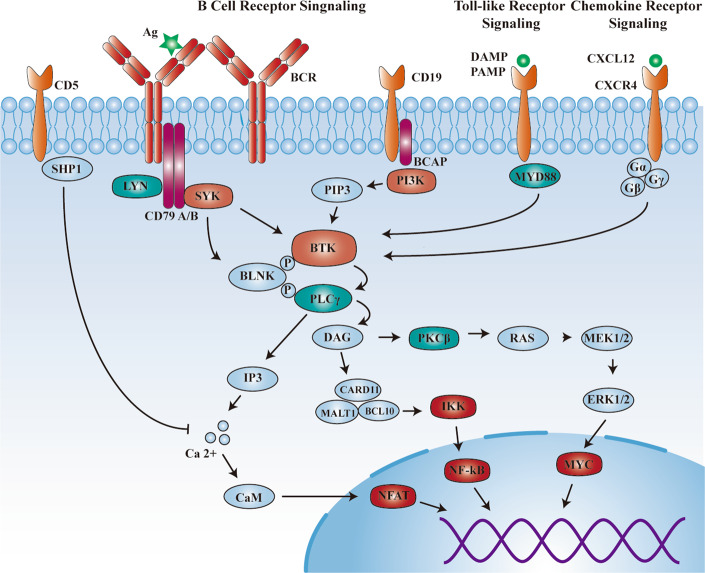
A schematic map of BTK in BCR signalling, TCR signalling, and chemokine receptor signalling pathway. BTK is a crucial component of antigendependent BCR signaling that regulates B cell proliferation and survival. Meanwhile, BTK also participates in antigen-independent Toll-like receptor signaling and chemokine receptor signaling, regulates B cell adhesion, migration, and tumor microenvironment forces.

In antigen-dependent BCR signalling, BTK can be activated by PI3K or SYK. BCR is always coupled with the Igα/Igβ (also known as CD79a/b) heterodimer, which is composed of the BCR complex. When specific antigens bind to the BCR complex, the SRC family LYN phosphorylates the immunoreceptor tyrosine-based activation motif residues on the cytoplasmic tails of CD79a/b and activates SYK. Activated SYK promotes B-cell linker protein (BLNK) to recruit and phosphorylate downstream effectors BTK and PLCγ2, and it can also phosphorylate tyrosine residues in the cytoplasmic tail of CD19, which can activate PI3K. Moreover, the cytoplasmic B-cell adapter for PI3K (BCAP) can also recruit PI3K^31^. PI3K generates an essential messenger for activating downstream pathways, named PIP3, which often binds with the PH domain of BTK and allows SYK and LYN to activate BTK by full transphosphorylation of Y551 site. In the negative feedback regulation, LYN phosphatases SH2 domain-containing inositol-5′-phosphatase-1 (SHIP1), which subsequently inactivates substrate proteins such as BTK, thereby inhibits the BTK membrane association. Downstream of BCR signalling, the primary substrate of BTK is PLCγ2. BTK phosphorylates PLCγ2 at positions Y753 and Y759, generating 2 s messengers including inositol triphosphate (IP3) and DAG, thus activating several signalling pathways. IP3 is involved in regulating intracellular Ca^2+^ levels, thereby activating T-cell transcription factors via calmodulin. DAG mediates the activation of PKCβ, which induces RAS signalling-dependent phosphorylation of ERK1/2. Importantly, PKCβ also activates the NF-κB pathway through a scaffold complex that includes caspase recruitment of CARD11, BCL 10, and MALT1, thereby regulating B-cell survival, proliferation, differentiation, and antibody secretion (Fig. 3) [6].

In chemokine receptor signalling, CXCL12, which is highly expressed in the bone marrow and germinal centres, can bind with CXCR4 and induce BTK activation by direct interactions between BTK and the CXCR4-linked heterotrimeric G protein subunits. G proteins contain αβγ subunits. Both Gα and Gβγ subunits can directly bind to BTK in the PH domain and TH domain. In addition, Gβγ subunits can also bind to the catalytic domain of BTK. Subsequently, activated BTK phosphorylates PLCγ2, ERK1/2, JNK, and AKT, thus regulating cell adhesion and migration [7].

In antigen-independent TLR signalling, most TLRs recruit MYD88 in response to the TLR ligand bacterial lipopolysaccharide. BTK can directly interact with five different molecules: the intercellular domains of most TLRs, the downstream adaptors MYD88 and MYD88 adaptor-like protein (MAL), IL-1R-associated kinase 1 (IRAK1) and TIR-domain-containing adapter-inducing interferon-β (TRIF), thus inducing the downstream transcription of NF-κB, activator protein 1 (AP1) and interferon regulatory factor (IRF3) activation, which promotes cell proliferation, antibody secretion, class switch recombination and the production of pro-inflammatory cytokines [7, 8].

### History of the discovery and characteristics of BTK inhibitors

Ibrutinib is a first-in-class inhibitor of BTK. After the first failed BTK inhibitor LFM-A13 was invented in 1999 [9], the compound PCI-32765, which was designed by Celera Genomics scientists, was initially chosen for preclinical development of in vivo models of rheumatoid arthritis in 2007 [10]. The efficacy of ibrutinib in B-cell lymphoma was first reported by Honigberg et al. [11]. The results showed that orally administered ibrutinib induced a response in three out of eight dogs with spontaneous B-cell non-Hodgkin lymphoma. Subsequently, to overcome off-target side effects and the emerging resistances of ibrutinib, some selective second-generation BTK inhibitors were developed. Acalabrutinib, also known as ACP-196, is a novel second-generation BTK inhibitor, which was designed by Acerta Pharma [12]. Similar to ibrutinib, Harrington et al. elected a canine model of B-cell NHL to evaluate the pharmacodynamic effects of acalabrutinib in vitro and vivo [13]. It demonstrated that acalabrutinib potently inhibited BTK activation, thus inhibiting the proliferation of CLBL1 cells, a canine B-cell lymphoma cell line. The overall response rate (ORR) was 25%, with a median PFS of 22.5 days in 20 dogs. Zanubrutinib, also known as BGB-3111, is a next-generation BTK inhibitor developed by BeiGene in 2012 [14]. It was designed with the concept of a structure–activity relationship-driven drug design strategy, and compound 31a synthesized in a series of pseudo-pyrimidinone compounds was selected as a potential candidate due to its high potency, selectivity, pharmacokinetics in vitro, and excellent pharmacodynamics in an OCI-LY10 DLBCL xenograft model [15].

There are similarities and differences among these three approved BTK inhibitors (Table 1): all inhibitors are irreversibly covalently bound to cysteine 481 in the ATP binding pocket of BTK; ibrutinib is the most potent BTK inhibitor followed by zanubrutinib and acalabrutinib based on biochemical binding kinetics, but differences in biochemical potency were partly lost in cellular assays using human peripheral blood mononuclear cells or human white blood cells (all less than 10 nM); acalabrutinib had the lowest off-target rate and the highest selectivity followed by zanubrutinib and ibrutinib [16]. The difference in pharmacodynamics and pharmacokinetics may influence the dosage, efficiency, and adverse events (AEs) of inhibitors in clinical practice. Acalabrutinib had a shorter half-life than ibrutinib, which is administered once a day, and the BTK occupancy was higher with twice daily than with once-daily dosing (95.3% vs 87.6%), which means that the drug requires twice daily dosing [17]. In zanubrutinib, sustained complete inhibition with more than 95% BTK occupancy in lymph nodes was more frequent with 160 mg twice daily than with 320 mg once daily. Therefore, 160 mg twice daily was selected as a recommended dose for further investigation [18]. A balance between fast absorption and fast elimination can bring rapid target inhibition and reduce the potential risk of off-target issues or drug interactions. The shorter half-life and selective properties of acalabrutinib allowed it to achieve complete and continuous inhibition of BTK without increasing the toxic effects from inhibition of alternative kinases. Full target coverage may reduce drug resistance caused by mutations in the BTK enzyme and may also lower the rate of Richter’s transformation [19]. Notably, acalabrutinib showed almost no inhibitory activity on EGFR, which is thought to be associated with rash and severe diarrhoea [20]; IL2-inducible T-cell kinase (ITK), which is critical for natural killer cell-mediated cytotoxicity, particularly antibody-dependent cell-mediated cytotoxicity (ADCC) [21]; or TEC kinase, which contributes to platelet dysfunction and the increases risk of bleeding [22]. It only inhibits BTK, BMX kinase, and human epidermal growth factor receptor 4 at clinically relevant concentrations, possibly contributing to the higher specificity and well-tolerated performance. Zanubrutinib is similar to acalabrutinib with less activity on TEC and ITK [23]. In summary, although there are some differential phenotypes in vitro among these three inhibitors, whether these diversities could translate into a clinical benefit and which BTK inhibitor is the best-in-class drug remain to be seen in subsequent head-to-head randomized clinical trials.

**Table 1:** Comparison of features and properties between ibrutinib, acalabrutinib, and zanubrutinib.

	Ibrutinib	Acalabrutinib	Zanubrutinib
Alternative names	PCI-32765	ACP-196	BGB-3111
Mechanism of action	C481, ATP-binding domain	C481, ATP-binding domain	C481, ATP-binding domain
Dosage	420 mg, qd (CLL/SLL, WM); 560 mg, qd (MCL, MZL)	100 mg, bid (MCL, CLL/SLL)	160 mg, bid (MCL)
Pharmacodynamics			
Kinetics (nM)	54.2	181	126
BTK (IC50, nM)	1.5	5.1	0.5
TEC (IC50, nM)	10	126	44
ITK (IC50, nM)	4.9	>1000	50
TXK (IC50, nM)	2	368	2.2
BMX (IC50, nM)	0.8	46	1.4
EGFR (IC50, nM)	5.3	>1000	21
ERBB2 (IC50, nM)	6.4	~1000	88
ERBB4 (IC50, nM)	3.4	16	6.9
BLK (IC50, nM)	0.1	>1000	2.5
JAK3 (IC50, nM)	32	>1000	1377
hPBMC (EC50, nM)	0.6	2.9	0.9
hWB (EC50, nM)	5.8	9.2	2.4
Pharmacokinetics			
Cmax (ng/ml)	35	323	346
tmax (h)	1–2	0.75	2
AUC (ng h/ml)	708	1111	1405
Vd,ss/F	10000	101	881
t1/2 (h)	4–6	0.9	3.31
Clearance (L/h)	62	159	182

### BTK inhibitors in MCL

#### Preclinical development of BTK inhibitors in MCL

In primary MCL cell lines and tissues, several kinases such as SYK, PI3K, LYN, and BTK are highly overexpressed and are correlated with NF‑κB activity, thus promoting the proliferation of MCL cells. Ibrutinib can diminish viability, and impaired CXCR4 or CXCR5 mediates adhesion and migration in vitro [24]. Blocking BCR signalling represents a promising approach for MCL.

#### Clinical development of BTK inhibitors in MCL

Advani et al. reported a phase 1 study that ibrutinib induced a response in 7/9 patients with refractory or relapsed (R/R) MCL and established 560 mg as the recommended Phase 2 dose (RP2D) [25]. PCYC-1104 study showed that ibrutinib is less toxic and more effective than the available intensive chemotherapy regimens, such as ESHAP, MINE, hyper-CVAD, and R-ICE (Table 2) [26]. Based on this study, ibrutinib was accelerated approved by the FDA for the treatment of R/R MCL in 2013. A randomized RAY study confirmed that ibrutinib is superior to temsirolimus in 280R/R MCL patients [27]. Temsirolimus was the unique chemotherapy-free therapy approved for R/R MCL in the European Union at that time. It also suggested that AEs such as atrial fibrillation, bleeding, and pneumonia are ibrutinib-related AEs. A pooled analysis of 370 patients in the RAY, SPARK, and PCYC-1104 studies demonstrated that the median times to first response and the best response were 2.07 and 2.14 months, respectively. The use of ibrutinib after the first relapse rather than later was associated with significant improvements in both progressive-free survival (PFS) and overall survival (OS). The prevalence of infection, diarrhoea, and bleeding was the highest for the first six months of therapy and less thereafter. The incidence of treatment-related grade 3–5 AEs of fibrillation and bleeding was 4.6% and 4.9%, respectively [28]. The ORR of ibrutinib (66%) in R/R MCL appears higher than that of other single-agent chemotherapy-free therapies, such as bortezomib (33%), lenalidomide (28%), temsirolimus (47%), ofatumumab (8%), obinutuzumab (27%), and idelalisib (40%), and it creates a possibility to develop an era of chemotherapy-free treatment for the management of MCL [29, 30]. By contrast, single-agent acalabrutinib demonstrated an ORR/CR of 81%/42%, and the FDA has accelerated approved it to treat R/R MCL based on this phase 2 trial in 2017 [31]. Zanubrutinib showed an ORR/CR of 86.5%/29.7% in a global phase I study and an ORR/CR of 84%/68.6% in the Chinese populations [32, 33]; furthermore, the FDA has also accelerated and approved it for R/R MCL in 2019. This suggests that the response rate of the same drug in the different races may vary, and infections were more frequent in Chinese patients. Notably, although the ORR/CR appears higher with acalabrutinib or zanubrutinib than with ibrutinib, patients in trials of acalabrutinib and zanubrutinib were exposed at an earlier line of treatment, and the Lugano 2014 criteria may potentially increase response rates.

**Table 2:** Current clinical trials of Bruton tyrosine kinase inhibitors in haematological malignancies and the latest extended follow-up results.

Trials	Intervention	Comparison	Numbers	Median age	ORR	CR	Median PFS	Median OS	Study design (Phase)
R/R CLL/SLL									
O’Brien et al. [53] (PCYC-1103)	Ibru		101	64	89%	10%	51 mon; 5 years: 44%	NR	Ib/II, 5 years extended follow-up
Munir et al. [55] (RESONATE, PCYC-1112)	Ibru	Ofatumumab	195 vs 196	67 vs 67	91% vs 11%	Ibru: 10.8%	44.1 vs 8.1 mon	67.7 vs 65.1 mon	III, 6 years extended follow-up
Jain et al. [56]	Ibru-R		40	65	95%	23%	45 mon	NR	II, 47 months extended follow-up
Brown et al. [62] (PCYC-1108)	Ibru-BR		30	62	97%	40%	12 mon:86.3%, 36 mon:70.3%	N/A	Ib
Fraser et al. [63] (HELIOS)	Ibru-BR	Placebo-BR	289 vs 289	64, 63	87.2% vs 66.4%	38.1% vs 8%	NR vs 14.3 mon; 36 mon: 68% vs 13.9%	NR	III, 34.8 months extended follow-up
Jaglowski et al. [57]	Ibru- Ofatumumab	Concurrent start; Ofatumumab lead-in	27 vs 20 vs 24	64	100% vs 79% vs 71%	N/A	12 mon: 89% vs 85% vs 75%	N/A	Ib/II
Hillmen et al. [58] (CLARITY)	Ibru-Venetoclax		47	64	89%	51%	N/A	N/A	II
Byrd et al. [59, 77] (ACE-CL-001)	Acala		134	66	94%	4%	N/A	N/A	I/II, 41 months extended follow-up
Awan et al. [79]^a^	Acala		33	64	76%	3%	N/A	N/A	II, 18.5 months extended follow-up
Ghia et al. [82] (ASCEND)	Acala	IR or BR	155 vs 155	N/A	81% vs 75%	0% vs 1%	NR vs 16.5 mon; 12 mon-PFS:88% vs 68%	20 mon: 94% vs 91%	III
Xu et al. [83]	Zanu		91	61	91%	4%	1 year: 80.9%	N/A	II
Naive CLL/SLL									
O’Brien et al. [53, 65](PCYC-1102)	Ibru		31	71	87%	29%	5 years: 92%	5 years: 92%	Ib/II, 5 years extended follow-up
Burger et al., [66] (Resonate-2)	Ibru	Chlorambucil	136 vs 133	73 vs 72	Ibru: 92%	Ibru: 30%	5 years: 70% vs 12%	5 years: 83% vs 68%	III, 5 years extended follow-up
Woyach et al. [70]	Ibru	Ibru-R, BR	182 vs 182 vs 183	71 vs 71 vs 70	93% vs 94% vs 81%	7% vs 12% vs 26%	NR vs NR vs 43 mon	2 years: 90% vs 94% vs 95%	III
Davids et al. [75]	Ibru-FCR		85	55	96%	36%	2 years: 100%	2 years: 100%	II
Moreno et al. [76] (iLLUMINATE)	Ibru- obinutuzumab	Chlorambucil - obinutuzumab	113 vs 116	70 vs 72	100% vs 85%	19% vs 8%	NR vs 19 mon; 30 mon: 79% vs 31%	N/A	III
Shanafelt et al. [52] (E1912)	Ibru-R	FCR	354, 175	56.7 vs 56.7	95.8% vs 81.1%	17.2%vs 30.3%	3 year: 89.4% vs 72.9%	3 years: 98.8% vs 91.5%	III
Sharman et al. [81] (ELEVATE TN)	Acala, Acala+ obinutuzumab	Obinutuzumab+ chlorambucil	179 vs 179 vs 177	N/A	85.5% vs 93.9% vs 78.5%	1% vs 14% vs 5%	NR vs NR vs 22.6 mon; 2 year: 87% vs 93% vs 47%	2 years: 95% vs 95% vs 92%	III
Naive and R/R CLL/SLL									
Woyach et al. [80]	Acala		19 vs 22	61 vs 63	95% vs 92%	N/A	39 mon: 94.4% (Naive); 42 mon: 72.7% (R/R)	39 mon: 100% (Naive;) 42 mon: 82% (R/R)	Ib/II
Cull et al. [113] ASH	Zanu		22 vs 98	67	100% vs 95.9%	13.6%vs 14.3%	2 year: 95% vs 88%	N/A	I/II, 25.1 months extended follow-up
Tam et al. [18, 32]	Zanu-obinu		45	68	96%	27%	N/A	N/A	Ib
High risk (del 17p, TP53 mutation) naive or R/R CLL/SLL									
Ahn et al. 2015, [72]	Ibru naive	R/R	35 vs 16	62 vs 62	96%	0%	5 year: 74.4% vs 19.4%	5 years: 85.3% vs 53.7%	II, 4.8 years extended follow-up
O’Brien et al. [73] (RESONATE-17)	Ibru		154	64	83%	10%	24 mon: 64%	24 mon:74%	II, 27.6 months extended follow-up
Sun et al. [114]	Acala 100 mg BID	Acala 200 mg QD	48	N/A	79.2% vs 85.8%	N/A	24 mon: 87.2% vs 91.5%	N/A	II
Tam et al. [18, 32] ASH (SEQUOIA)	Zanu		109	70	92%	0%	N/A	N/A	III
R/R MCL									
Wang et al. 2015, [26] (PCYC-1104-CA)	Ibru		111	68	67%	23%	Median: 13 mon	Median: 22.5 mon	II, 26.7 months extended follow-up
Rule et al. [115] (RAY)	Ibru	Temsiromlimus	139 vs 141	67 vs 68	77% vs 47%	23% vs 3%	15.6 vs 6.2 mon	30.3 vs 23.5 mon	III, 3 years extended follow-up
Jain et al. 2016, [41]	Ibru-rituximab		50	67	88%	58%	43 mon; 3 year: 54%	NR; 3 years: 69%	II, 47 months extended follow-up
Tam et al. [116]	Ibru-venetoclax		24	68	76%	57%	12 mon: 75%; 18 mon: 57%	12 mon: 79%; 18 mon: 74%	II
Jerkeman et al. [43] (PHILEMON)	Ibru-Len-rituximab		50	69	75%	56%	16 mon	20 mon	II
Martin et al. [39]	Ibru-palbociclib		27	65	67%	37%	2 years: 59.4%	2 years: 60.6%	I
Wang et al. [31, 46] (ACE-LY-004)	Acala		124	68	81%	42%	20 mon; 24 mon: 49%	24 mon: 72.4%	II, 26 months extended follow-up
Tam et al. [18, 32]	Zanu naive	R/R	45	71	87.5% vs 86.5%	37.5%vs 29.7%	R/R 14.7mon	N/A	I
Song et al. [33]	Zanu		86	60.5	84%	68.6%	22.1 mon	N/A	II
Naive MCL									
Wang et al. [46] Lugano (WINDOW -1)	Ibru-RCVAD		50	N/A	100%	92%	3 years: 88%	3 years: 100%	II
Naive and R/R WM									
Treon et al. [87]^b^	Ibru		63	63	91%	73%	2 years: 69.1%	2 years: 95.2%	II
Dimopoulos et al. [117] (INNOVATE)	Ibru-R	Placebo-R	75 vs 75	70 vs 68	92% vs 47%	72% vs 32%	NR vs 20.3 mon; 24 mon: 80% vs 37%	NR vs NR; 30 mon: 94% vs 92%	III
Treon et al. [88]^c^	Ibru		30	67	100%	83%	18 mon: 92%	N/A	II
Owen et al. [89]	Acala naive	R/R	14 vs 92	73 vs 69	93% vs 93%	0% vs 0%	2 years: 90% vs 82%	2 years: 92% vs 89%	II
Trotman et al. [118] EHA	Zanu		24 vs 49	67	96% vs 90%	0% vs 2%	2 years: 81%	N/A	I
Dimopoulos et al. [119] (ASPEN cohort 2)	Zanu naive	R/R	5, 21	N/A	80% vs 76.2%	0% vs 0%	N/A	N/A	III
Tam et al. [120] (ASPEN cohort1)	Zanu	Ibru	102 vs 99	N/A	28.4% vs 19.2%	N/A	1 year: 89.7% vs 87.2%	1 year: 97% vs 93.9%	III
R/R MZL									
Noy et al. [90]	Ibru		63	66	48%	3%	14.2 mon	18 mon: 81%	II
R/R DLBCL									
Wilson et al. [94]	Ibru		80	64	37%	10%	1.64 mon	6.41 mon	I/II
Maddocks et al. [40]	Ibru-BR		16	62	37%	31%	N/A	N/A	I
Sauter et al. [95]	Ibru-R-ICE	GCB/Non-GCB/PMBL	3 vs 8 vs 4	59	33% vs 100% vs 100%	0% vs 0% vs 100%	N/A	N/A	I
Goy et al. [91]	Ibru-Len-R	non-GCB	23	64	65%	41%	N/A	N/A	I
Younes et al. [96, 104]	Ibru-Nivo		45	64	36%	16%	2.6 mon	N/A	I/IIa
Naive DLBCL									
Younes et al. [121]	Ibru-RCHOP		18	61	100%	83%	N/A	N/A	I
Younes et al. [96, 104] (non-GCB)	Ibru-RCHOP	Placebo-RCHOP	419 vs 419	63 vs 61	89.3% vs 93.1%	67.3% vs 68%	36 mon: 70.8% vs 68.1%	36 mon: 82.8% vs 81.4%	III
R/R PCNSL									
Grommes et al. [122]	Ibru		13	69	77%	37%	N/A	N/A	I
Soussain et al. [97]	Ibru		52	67.5	52%	19%	4.8 mon	19.2 mon	II
R/R FL									
Bratlett et al. [123] (P2C)	Ibru		40	64	38%	13%	14 mon; 2 years: 20.4%	2 years: 79%	II
Gopal et al. [99] (DAWN)	Ibru		110	61.5	21%	11%	4.6 mon	12 mon: 78%; 30-mon: 61%	II
Tam et al. [18, 32]	Zanu-obinu		36	59	72%	36%	N/A	N/A	Ib
Naive FL									
Ujjani et al. [124]	Ibrutinib-Len-R		22	53.5	95%	36%	N/A	N/A	I
R/R MM									
Richardson et al. [103]	Ibru		92	65	28%	N/A	4.6 mon	N/A	II
Chari et al. [102]	Ibru-carfilzomib- dexamethasone		43	63	76%	2%	20.5 mon	NR	I

*ORR* overall response rate, *CR* complete response, *PFS* progressive-free survival, *OS* overall survival, *AE* adverse events, *Ibru* Ibrutinib, *Acala* acalabrutinib, *Zanu* zanubrutinib, *B* bendamustine, *R* rituximab, *Len* lenalidomide, *FCR* fludarabine-cyclophosphamide-rituximab, *ICE* ifosfamide-carboplatin-etoposide, *Obinu* obinutuzumab, *Nivo* nivolumab, *RCHOP* rituximab-cyclophosphamide-doxorubicin-vincristine-prednisone, *CVAD* cyclophosphamide-vincristine-doxorubicin-dexamethasone, *mon*, Months, *N/A* not available, *NR* not reach, *R/R* refractory or relapse.

^a^Included R/R CLL who intolerant to ibrutinib.

^b^Only included R/R WM.

^c^Only included Naive WM.

The mortality rate and drug resistance are significantly increased in MCL patients who did not achieve a complete response (CR) after first-line treatment. During long-term follow-up, the rate of a CR of single-agent ibrutinib in MCL was 20%, but the 2-year PFS and OS rate were 79% and 92%, respectively, in patients who achieved a CR with ibrutinib. This evidence would encourage combining ibrutinib with other regimens in an attempt to maximize the CR rate. In R/R MCL, preclinical models indicate that inhibition of both BTK and BCL2 is synergistic, and both the BTK inhibitor ibrutinib and the BCL2 inhibitor venetoclax achieved an ~21% CR rate for each agent [34–36]. In consideration of the different mechanisms and minor overlapping toxicities, the combination of these two drugs may improve efficacy. Tam et al. conducted a phase 2 study, and it demonstrated a CR rate of 59% at 4 months. MCL is characterized by cell-cycle dysregulation, and the CDK4 inhibitor palbociclib can prolong early G1 cell arrest in MCL tumour cells [37]. The combination of ibrutinib and palbociclib synergistically killed ibrutinib-resistant MCL cells in vitro, probably by inhibition of compensatory signalling pathways, such as PI3k signalling [38]. A phase 1b trial reported that this combination yielded a CR rate of 37% in R/R MCL [39]. A subsequent phase 2 multicentre trial to further characterize the efficacy of this combination is now ongoing (NCT03478514). Furthermore, ibrutinib in combination with bendamustine/rituximab (BR) showed a 94% ORR and 76% CR rate [40]. The ORR/CR rates of BR, ibrutinib/rituximab (IR), rituximab/lenalidomide, IR/lenalidomide were 82%/40%, 88%/58%, 57%/36% and 75%/56%, respectively [41–45]. It is unknown whether this triplet combination will translate into a longer PFS/OS than achieved with a single- or two-agent combination at present. However, this will probably be further addressed in the upcoming phase 3 randomized trial (SHINE, NCT01776840) evaluating first-line BR with or without ibrutinib in treatment-naive patients aged ≥65 years who are not eligible for transplant.

The exploration of ibrutinib as a first-line option had considerable success in recent years but needs further confirmation, and the combination of chemotherapy-free agents may replace the standard toxic chemotherapies in the next 5 years. For young patients (≤65 years), WINDOW-1 showed that chemotherapy-free induction with IR followed by 4 cycles of R-hyper-CVAD consolidation is extremely potent and safe [46]. For elderly patients (≥65 years), the IR combination is highly effective [47].

### BTK inhibitors in CLL/SLL

#### Preclinical development of BTK inhibitors in CLL/SLL

In CLL cells, constitutive phosphorylation of LYN, SYK, PKCβ, BTK, and PI3K and activation of NF‑κB could interact with microenvironmental stimuli, therefore initiating or maintaining the survival, proliferation or migration of CLL cells [48]. Lyn, Btk, Pkcβ, or Nf-κB p50 deficient in Eμ-TCL1 mice, a CLL-like mouse model, significantly delayed the onset and reduced the burden of leukaemia but still initiated lymphomagenesis. BTK deficiency in vivo abrogated tumour formation, whereas overexpression of BTK increased tumour incidence and overall mortality [49]. Regarding dynamic interactions between CLL cells and their microenvironment, macrophages in lymphoid organs exhibit M2-like phenotype nursing CLL cell survival and proliferation, while ibrutinib may disrupt this interaction [50]. Zanubrutinib also induced favourable changes in the immune microenvironment by improving T-cell exhaustion and downregulating checkpoint molecules on suppressor cells and adhesion/homing receptors on B cells [51]. These clues suggested that BTK inhibitors exert substantial effects on the B-cell malignancy microenvironment.

#### Clinical development of BTK inhibitors in CLL/SLL

CLL/SLL accounts for ~40% of all adult leukaemias and 11% of haematologic neoplasms. It often occurs in older people, with a median age of 72 years. Although previously the chemotherapy fludarabine-cyclophosphamide-rituximab (FCR) combination appeared effective, it is associated with substantial toxic effects such as severe myelosuppression and infectious complications [52]. Ibrutinib offers a chemotherapy-free treatment option with an acceptable side effect profile. The exploration was started in R/R CLL/SLL patients. The PCYC-1103 study established 420 mg as the RP2D, and the randomized phase III RESONATE trial (PCYC-1112) proved that ibrutinib is superior to anti-CD20 ofatumumab, even in patients with unfavourable risks, such as del 17p, del 11q, and unmutated IGVH [53–55]. Based on these findings, the FDA approved ibrutinib for CLL/SLL patients who have received at least one prior therapy in 2014.

Because single-agent ibrutinib has shown good tolerability, several studies have sought to combine ibrutinib with other chemoimmunotherapy regimens, such as rituximab, ofatumumab, venetoclax, or bendamustine and rituximab (BR) in R/R CLL/SLL to improve the efficacy further [56–58]. The utility of rituximab plus ibrutinib (IR) has been called into question given that the reported PFS is very close to what has been reported with the use of single-agent ibrutinib [54, 59]. Other studies also reported decreased ADCC with rituximab in vivo as well as downregulation of CD20 in CLL B cells following ibrutinib treatment [60]. Ongoing studies randomizing R/R CLL patients to either ibrutinib treatment alone or combined ibrutinib–rituximab treatment should help to clarify this question. Ibrutinib has been reported to affect the ADCC of ofatumumab less than that of rituximab. This has led to the development of combination strategies of both substances in a phase Ib/II study. Jaglowski et al. demonstrated that ibrutinib lead-in seems more powerful than a concurrent start or ofatumumab lead-in sequence [57]. Moreover, PCYC-1108 and the randomized phase III HELIOS trial compared ibrutinib plus standard chemoimmunotherapy with BR to BR and suggested that ibrutinib-BR was superior to the placebo-BR; however, whether the efficacy of ibrutinib-BR is superior to single-agent ibrutinib in CLL/SLL patients need further discussion [61–63].

Can ibrutinib work as a first-line therapy or high-risk CLL/SLL populations? PCYC-1102 demonstrated that ibrutinib yielded a high ORR (87%) in untreated CLL/SLL [64–66]. Although 20% of patients had a partial remission with lymphocytosis, generally in the first weeks of treatment, it is believed that this was due to the redistribution of CLL cells from solid lymphoma manifestations into the bloodstream and should not be confused for disease progression [67]. The phase III RESONATE-2 trial showed that ibrutinib resulted in a significantly longer PFS and OS than did chlorambucil in previously untreated older CLL/SLL patients [66, 68]. Since the German CLL-11 trial has proven that treatment with obinutuzumab-chlorambucil is superior to chlorambucil monotherapy [69], the choice of chlorambucil as a parallel arm in the RESONATE-2 study would be less informative than obinutuzumab-chlorambucil as a comparison. In addition, Woyach et al. conducted a phase III trial and suggested that treatment with ibrutinib was superior to treatment with BR, and there was no significant difference between ibrutinib and IR with regard to PFS [70]. According to these findings, the FDA approved ibrutinib alone as first-line therapy in CLL/SLL in 2016. A pooled analysis of PCYC-1102 and PCYC-1112 showed that patients receiving ibrutinib as first-line therapy and those without bulky disease had a better likelihood of a CR with treatment, and the median PFS and OS were longer in R/R patients who were treated with fewer prior therapies [71]. Del 17p or TP53 mutations are well established to cause poor sensitivity to classical immunochemotherapy, resulting in early relapse and short survival. The RESONATE-17 trial and Ahn et al. focused on this subset specifically and proved that ibrutinib performed well in CLL/SLL, irrespective of treatment history or genomic features [72–74].

First-line ibrutinib combinations have also been attempted by clinicians. Ibrutinib plus FCR showed an ORR of 96% with a 36% CR or CRi [75]. The iLLUMINATE trial showed that the median PFS was significantly longer with the chemotherapy-free ibrutinib–obinutuzumab regimen than chlorambucil–obinutuzumab [76]. On April 21, 2020, the FDA approved IR for naive CLL/SLL based on the phase III E1912 trial, which compared the efficacy between IR with standard chemoimmunotherapy FCR [52].

Given the success of ibrutinib, several clinical trials were directly conducted both in naive and R/R CLL/SLL patients to determine whether the second-generation irreversible BTK inhibitors acalabrutinib and zanubrutinib would be effective. Byrd, Awan, and Woyach et al. reported that acalabrutinib showed an approximate ORR of 90% in ibrutinib-treated or not treated, naive or R/R CLL/SLL, although the longest follow-up time was only 41 months and data maturity requires time [77–80]. These interim analyses demonstrated favourable safety and durability of the response with acalabrutinib, leading to FDA approval of acalabrutinib as a treatment for R/R CLL/SLL in 2019. ELEVATE-TN reported that acalabrutinib alone or acalabrutinib–chlorambucil is superior to obinutuzumab–obinutuzumab [81]. ASCEND reported that acalabrutinib improved PFS compared with idelalisib–rituximab or BR [82]. Several phase III clinical trials of acalabrutinib in CLL/SLL are ongoing, including ELEVATE-RR, which compares acalabrutinib to ibrutinib, and ACE-CL-311, which compares acalabrutinib-venetoclax with/without obinutuzumab versus chemoimmunotherapy. A phase I/II dose-escalation exploration trial of zanubrutinib found that 160 mg twice daily possessed a higher BTK occupancy in lymph node biopsy specimens than 320 mg once daily. The dose expansion cohort showed an approximate ORR of 100% [18]. In 116 Chinese R/R CLL/SLL patients, the ORR was 91.2% [83]. Other trials, such as a phase Ib study assessing zanubrutinib-obinutuzumab and the phase III SEQUOIA (BGB-3111-304) trial evaluating zanubrutinib in untreated CLL/SLL patients with Del 17p, are ongoing.

### BTK inhibitors in WM

#### Preclinical development of BTK inhibitors in WM

WM is characterized by high levels of monoclonal immunoglobulin M secreted by lymphoplasmacytic lymphoma cells with bone marrow infiltration. In the past, the anti-CD20 monoclonal antibody rituximab alone or combination therapies, such as rituximab-cyclophosphamide-dexamethasone, rituximab-bendamustine, or rituximab-bortezomib-dexamethasone, is commonly used in frail patients or patients with immunologic complications. However, patients presenting with high baseline IgM levels are prone to suffer from rituximab-related IgM flare, and the population will inevitably become refractory to rituximab, leading to an imperative need for new therapeutic choices [84]. MYD88 and CXCR4 somatic mutations play an essential role in the pathogenesis of WM. Approximately 91% of WM with MYD88 L265P mutations has constitutively activated BTK and NF-κB pathways. Approximately 30% of WM patients have CXCR4 mutations. CXCR4 mutations contain nonsense mutations and frameshift mutations [85]. CXCR4 activation promotes AKT kinase and ERK function, which may be associated with resistance to BTK inhibition. Ibrutinib can abrogate MYD88 L265P–BTK association, reduce NF‑κB activation, and induce apoptosis in WM cells [86].

#### Clinical development of BTK inhibitors in WM

The clinical activity of ibrutinib in WM was observed in a phase I study initially [25]. Treon et al. reported that the ORR and major response rates were highest among patients with MYD88^L265P^CXCR4^WT^ (100% and 91.2%) followed by MYD88^L265P^CXCR4^WHIM^ (85.7% and 61.9%) and MYD88^WT^CXCR4^WT^ (73.4% and 28.6%) [87]. By indirect comparison with other WM therapies, with ORRs of 40 to 80% and median PFS rates of 8 to 20 months, ibrutinib had accelerated approval by the FDA for the treatment of R/R WM patients in 2015 [87]. Later, the iNNOVATE study showed that IR was better than placebo plus rituximab both in untreated and R/R WM patients. Therefore, the FDA approved ibrutinib as a first-line treatment for WM in 2018. Treon et al. suggested that ibrutinib is highly active in untreated patients with symptomatic WM, especially in CXCR4 wild-type patients [88]. Several novel combinations of ibrutinib with BR(NCT01479842), lenalidomide (NCT01955499), pembrolizumab (NCT03679624) and daratumumab (NCT03679624) are under clinical development. On the other hand, acalabrutinib showed an ORR of 93% in both untreated and R/R cohorts [89]. The 24-month PFS rate of zanubrutinib for WM was 81%. The phase III ASPEN trial directly compared zanubrutinib to ibrutinib. Unfortunately, no statistical significance was found in the primary endpoint.

### BTK inhibitors in MZL

MZL is a heterogeneous B-cell malignancy arising from the post-germinal centre marginal zone B cells; it is frequently linked to chronic infection, such as hepatitis C virus and Helicobacter pylori. Chronic infection may lead to antigen-mediated BCR activation, resulting in aberrant B-cell survival and proliferation. A phase II study conducted in all subtypes of MZL identified ibrutinib as a single active agent with a favourable toxicity profile, and the ORR was similar to that of another approved regimen; therefore, it was accelerated for approval for R/R MZL in 2017 [90]. A phase III clinical trial (SELENE study) evaluating ibrutinib versus placebo in addition to either BR or R-CHOP immunochemotherapy is currently ongoing with pending results (NCT01974440).

### BTK inhibitors in DLBCL

#### Preclinical development of BTK inhibitors in DLBCL

DLBCL is the most common NHL and is classified into three types by gene expression profiles: **germinal centre B-cell (GCB), activated B cell (ABC)**, and an unclassifiable subtype. ABC-DLBCL is mainly dependent on BCR signalling for survival and proliferation: approximately 10% of cases demonstrate mutations in CARD11, resulting in constitutive downstream activation of NF-kB; 20% of cases harbour mutations in the ITAM of CD79A and CD79B, resulting in downstream kinase activation of SYK, BTK, PI3K, and PKCβ; and 30% of cases have MYD88 L265P mutations that directly activate the NF-κB pathway. However, GCB-DLBCL relies primarily upon PI3K/AKT activation rather than NF-κB activation. This may be the reason why ibrutinib is more sensitive in ABC-DLBCL. In preclinical models, ibrutinib synergized with lenalidomide could kill ABC-DLBCL cells by downregulating IRF4 [91]; ibrutinib synergized with bortezomib can increase apoptosis in bortezomib-resistant DLCBL cells via AKT and NF-κB inactivation, downregulation of MCL1, Bcl-xL, XIAP-enhanced DNA damage, and endoplasmic reticulum stress [92]; the combination of ibrutinib and PD-L1 antibody enhanced the modest effects seen with PD-L1 inhibition alone, decreased tumour growth and increased survival even in models that were insensitive to ibrutinib or did not express BTK [93]. These results suggest that ibrutinib might have a role in modulating the immune system, possibly through its effect as an inhibitor of ITK, which plays a part in T-cell proliferation and differentiation.

#### Clinical development of BTK inhibitors in DLBCL

Wilson et al. reported that the median PFS was longer in ABC-DLBCL; the ORR in patients with CD79B mutations was higher than that in those with wild type; there was no significant difference between tumours with MYD88 mutations and those with wild type; tumours with both CD79B and MYD88 mutations were more responsive than those with CD79 wild-type and MYD88 mutations among ABC-DLBCL [94]. These data support the use of gene signature as a biomarker to identify ibrutinib-responsive subjects in followed trials. The combination of ibrutinib with standard chemotherapy BR, ifosfamide–carboplatin–etoposide (ICE), or lenalidomide–rituximab was effective in a certain extent but warrants further exploration [40, 91, 95]. The PHOENIX trial showed that ibrutinib plus rituximab–cyclophosphamide–doxorubicin–vincristine–prednisone (R-CHOP) compared with R-CHOP did not meet its primary endpoint in untreated ABC-DLBCL, and the increased toxicity lead to caution [96]. In the subgroup analysis, an improved PFS and OS were observed in patients aged younger than 60 years. The subgroup analysis of regional diversity is ongoing, but it appears that ibrutinib plus R-CHOP is more efficient in Chinese patients. This might be because the Chinese population has a distinct gene-phenotype, younger age, smaller tumour burden, and shorter time from diagnosis to treatment. The next mission for PHOENIX is to expand the samples in a cohort of younger patients because the statistical theory suggests that more than 500 patients are needed to verify the benefits of OS. Above all, the data of single-agent ibrutinib or combination regimens in DLBCL are limited. The added toxic effects and the risk:benefit ratio in the combination regimen should be cautiously balanced. Precise gene-phenotyping may also be of benefit in selecting ibrutinib-sensitive patients. Promising SMART START and ImbruVeRCHOP trials are pending.

### BTK inhibitors in primary central nervous system lymphoma (PCNSL)

PCNSL is an aggressive lymphoma manifesting in the CNS, and the pathological classification of PCNSL mostly belongs to DLBCL. Due to the blood–brain barrier, patients with R/R PCNSL respond poorly to the majority available therapies. Given the activating MYD88 and CD79B mutations in PCNSL, a phase II clinical trial showed favourable PFS benefits. The PFS of ibrutinib in this study was longer than that in other previous reported therapies, including the mTOR inhibitor temsirolimus (PFS 2.1 months), rituximab-temozolomide (PFS 1.6 months), and topotecan (PFS 2 months) [97]. In addition, the response rates in PCNSL were considerably higher than those reported for unspecified DLBCL. It was concluded that the brain microenvironment might augment BTK dependence through chronic antigen presentation and BCR activation.

### BTK inhibitors in FL

Both antigen-dependent and independent BCR activation are exhibited in FL. The tumour microenvironment may contribute to the development and progression of FL, and the interaction of FL cells with immune cells in the tumour may influence the clinical course and response to therapy. The phase II P2C and DAWM trials showed a 21–38% ORR in R/R FL [98, 99]. The response rate in rituximab-sensitive patients was higher than that in ibrutinib-resistant patients. A possible explanation is that patients responding to rituximab have intact immune functionality compared with those who are refractory, and this, in turn, has an impact on ibrutinib-mediated immunomodulation. Patients with CARD11 mutations were predicted to be resistant to ibrutinib. Regulatory T cells were downregulated after three cycles, and the Th-1 antitumour cytokines IFN-r and IL-12 were increased in responsive patients. In Alliance A051103, the triplet combination of rituximab–lenalidomide–ibrutinib in previously untreated FL showed no improvement in efficacy but had a high incidence of toxicity compared with the promising activity of rituximab and lenalidomide in untreated FL (ORR 90–96%) and ibrutinib in R/R FL (ORR 30–35%) [100]. Above all, the response rates of ibrutinib in the R/R FL did not seem to be as encouraging as those seen in other B-cell malignancies. Exclusion of patients with CARD11 mutations and rituximab-refractory disease from trials evaluating BTK inhibitors may be considered to enrich the findings for responders.

### BTK inhibitors in MM

MM is a malignancy of plasma cells that accumulate in the bone marrow and show a low rate of proliferation. MM cells originate from plasma cells that no longer express a BCR on their cell surface. In vitro, ibrutinib inhibits the receptor activator of NF-κB ligand-induced phosphorylation of BTK and downstream PLCγ2 and inhibits human osteoclast function. In osteoclasts or bone marrow stromal cells from patients with MM, ibrutinib downregulated the secretion of carcinogen-initiated chemokines and cytokines, including CCL3, TNFβ, APRIL, and CXCL12, blocked CXCL12-induced adhesion and migration of MM cells and reduced IL6-induced cell growth [101]. In clinical trials, ibrutinib plus dexamethasone or ibrutinib–carfilzomib–dexamethasone demonstrated encouraging responses with a manageable safety profile [102, 103].

### PD-1 antibody combined with BTK inhibitors in B-cell malignancies

Since the emergence of immune checkpoint inhibitors has transformed the treatment of several malignancies, preclinical studies have reported synergistic antitumour effects between ibrutinib and immune checkpoint blockade. The combination of ibrutinib and nivolumab had a manageable safety profile in B-cell malignancies, but the clinical activity was similar to that of single-agent ibrutinib or nivolumab in the previous studies [104]. The ORR of zanubrutinib–tislelizumab in GCB-DLBCL, ABC-DLBCL, Richter transformation, FL, and PCNSL were 33.3%, 40%, 50%, 35.7%, and 33%, respectively [105].

### BTK inhibitors in solid cancers

Beyond effects on B-cell lymphoma, ibrutinib led to vasculature collapse, anti-fibrotic effects, T-cell restoration, and tumour regression and showed a synergistic effect with standard gemcitabine to extend survival in a murine pancreatic ductal adenocarcinoma (PDAC) model. However, the RESOLVE trial showed that ibrutinib plus nab-paclitaxel/gemcitabine did not improve PFS and OS compared to placebo plus nab-paclitaxel/gemcitabine in PDAC (Table 3). The efficacy of acalabrutinib–pembrolizumab in platinum-refractory metastatic urothelial carcinoma or advanced pancreatic cancer is still lower than that of standard chemotherapy [106]. Recruitment of mast cells within the microenvironment of neuroendocrine neoplasms (NENs) has been shown to regulate neoangiogenic and macroscopic tumour expansion, and ibrutinib can inhibit the proliferation of NEN cells and induce tumour regression through the inhibition of mast cell degranulation in vitro. However, there were no patients who responded in the phase II study [107]. Preclinical data also have shown that the combination of PD-L1 antibody and ibrutinib suppresses tumour growth in mouse models of lymphoma, triple-negative breast cancer, and colon cancer, most likely due to inhibition of Itk on T cells and in a Btk-independent manner [93]. In a phase 1b/2 clinical study, the combination of ibrutinib with durvalumab had limited antitumour activity in R/R patients with advanced pancreatic, breast, or lung cancers [108].

**Table 3:** Current clinical trials of Bruton tyrosine kinase inhibitors in solid tumours.

Trials	Patients	Intervention	Comparison	Number	Median age	ORR	CR	Median PFS	Median OS	Study design
Tempero et al. 2019 (RESOLVE)	Pancreatic ductal adenocarcinoma	Ibru-PTX-Gem	Placebo-PTX-Gem	211 vs 213	N/A	29% vs 42%	N/A	5.3 vs 6 mon	9.7 vs 10.8 mon	III
Overman et al. [106]	Advanced pancreatic cancer	Acala	Acala- Pembrolizumab	35 vs 38	64 vs 64	0% vs 7.9%	0% vs 0%	1.4 vs 1.4 mon	3.6 vs 3.8 mon	II
Hong et al et al. [108]	Advanced pancreatic, breast, lung cancers	Ibru-durvalumab		122	60.5	2% vs 3% vs 0%	N/A	1.7 vs 1.7 vs 2 mon	4.2 vs 4.2 vs 7.9 mon	Ib
Al-Toubah et al. [109]	Neuroendocrine neoplasms	Ibru		20	N/A	0%	N/A	3 mon	24.1 mon	II
Overman et al. 2016	Urothelial carcinoma	Acala- Pembrolizumab	Pembrolizumab	40 vs 35	N/A	20% vs 26%	10% vs 9%	2.2 vs 1.6 mon	6.3 vs 11.4 mon	II

*ORR* overall response rate, *CR* complete response, *PFS* progressive-free survival, *OS* overall survival, AE adverse events, *Ibru* Ibrutinib, *mon* Months, *N/A* not available, *Gem* Gemcitabine, *PTX* nab-pacitaxel.

### AEs in approved BTK inhibitors

**Fig. 4:**
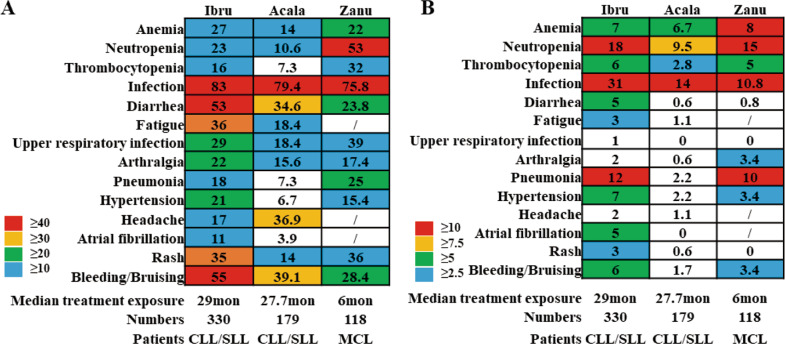
Frequency of adverse events with ibrutinib, acalabrutinib, and zanubrutinib. **A** The comparison of any grade adverse events in ibrutinib, acalabrutinib, and zanubrutinib. **B** The comparison of grade 3-5 adverse events in ibrutinib, acalabrutinib, and zanubrutinib.

The frequency of AEs with three BTK inhibitors were diverse (Fig. 4). Bleeding, infections, haemorrhage, atrial fibrillation, and headache are the most concerning AEs in clinical treatment. In the integrated analysis of RESONATE and RESONATE-2, the ibrutinib-related emergent AEs are infections, bleeding, and atrial fibrillation. Over time, the prevalence of most AEs trended down; the prevalence of hypertension was increased, but the incidence decreased after 1 year [109]. Acalabrutinib showed a similar incidence of infections and bleeding and a lower incidence of atrial fibrillation, but it easily causes headaches [81]. Zanubrutinib showed a higher incidence of haematologic AEs, but the reports of rash, atrial fibrillation, or bleeding were rare. In clinical practice, we can choose different BTK inhibitors, according to their differential toxicity performance. Acalabrutinib is not recommended for patients with headache. Ibrutinib is not recommended for patients who have a high risk of cardiovascular and cerebrovascular diseases, and zanubrutinib may be a better choice. The combination of BTK inhibitors with anticoagulants should be used with extreme caution.

Unlike other regimens for CLL that are given for a finite number of cycles, BTK inhibitors are prone to continue until PD or unacceptable AEs, leading to extended clinical benefit in most patients. Thus, the long-term toxicity in BTK inhibitors should be particularly concerning. Ibrutinib, which was first approved in 2013, has accumulated larger samples and longer follow-up data than other BTK inhibitors, which could benefit our clinical applications. Although indirect comparison of safety profiles of three BTK inhibitors was discussed, head-to-head randomized trials are warranted in the future.

### Drug resistance in BTK inhibitors

Although BTK inhibitors have been proven to be one of the most effective agents in several B-cell malignancies, cases of primary and secondary resistance have emerged and usually resulted in a poor prognosis. Emerging cases of resistance have underlined the need for clinical biomarkers to predict sensitivity or resistance to the BTK inhibitors. Mutated IGHV in WM or MYD88 mutations with CD79A/B wild type in ABC-DLBCL may be associated with primary resistance to ibrutinib [94]. Among CLL/SLL patients who progressed on ibrutinib, mutations in the ibrutinib binding (BTK Cys481), gatekeeper (BTK Thr474), and SH2 (BTK Thr316) domains of BTK have been discovered [110]. Cysteine to serine mutations at the C481 site permits downstream signalling, including PLCγ2 and CARD11, activation, hence bypassing the inactive BTK promoting distal BCR signalling activation, resulting in tumour cell proliferation, and migration. Metabolic reprogramming toward oxidative phosphorylation glutaminolysis is associated with therapeutic resistance to the ibrutinib in MCL [111]. BTK Cys481 mutations are common in WM patients with clinical progression on ibrutinib and are associated with mutated CXCR4 [112].

The existing three BTK inhibitors all target BTK at the C481 site; therefore, it may not be effective to switch to another BTK inhibitor when resistance occurs. Strategies to overcome acquired resistance may be concluded as follows: to develop next-generation non-covalent BTK inhibitors that do not interact with Cys481; to combine BTK inhibitors with PI3K, SYK, or BCL-2 inhibitors to inhibit the activation of bypass signalling; to treat with other novel therapies such as chimeric antigen receptor T-cell immunotherapies; and to rebiopsy and conduct sequencing therapy to select other appropriate target treatments.

### Global trends in BTK inhibitors

**Fig. 5:**
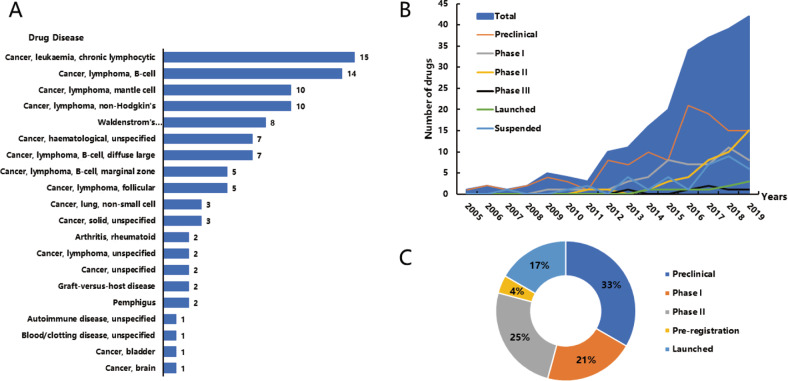
Global trends in BTK inhibitors. **A** The indication distribution of BTK inhibitors in clinical trials of cancers; **B** The landscape of BTK inhibitors between 2005–2019; **C** The global status of BTK inhibitors in clinical trials of cancers.

By searching published articles and the Pharmaproject database, we systematically reviewed changes over time in clinical trials of BTK inhibitors globally to provide insight on changes in the drug development process of BTK inhibitors and identify unmet clinical needs. A total of 87 new BTK projects focusing on cancer, arthritis, or other fields were initiated from 2005 to 2019, with a sharp increase after 2013 (Fig. 5). Up to June 2020, 24 BTK inhibitors are still developing in the cancer field (Supplementary Table 1), and half of them are in the clinical development phase. CLL/SLL, B-cell malignancies, and MCL are the top 3 indications for clinical trials of BTK inhibitors. The USA, China, and Poland are the top three countries for clinical trials. Although the efficacy of BTK inhibitors in DLBCL or solid tumours is still poor, and several inhibitors are still struggling for indications expansion. To brief summarize, BTK is an important target in the drug development field with fierce competition in ~24 ongoing congeneric products. Future development of BTK inhibitors should consider differentiation products either in terms of the indications for development or unusual action mechanisms. Improving the infiltration ability across the blood–brain barrier or solving BTK resistance may offer a breakthrough.

## Concluding remarks

BTK inhibitors are promising novel agents that have potential efficiency in B-cell malignancies and autoimmune diseases. In this review, we summarized a total of 73 clinical trials, including 48 trials published or updated with extended follow-up results in the recent 3 years up to June 2020, and the development process from bench to bedside of BTK inhibitors. Ibrutinib is the first-in-class BTK inhibitor and has been approved in more than 80 countries. The FDA has approved ibrutinib for CLL/SLL, WM, R/R MCL, R/R MZL, and R/R chronic graft-versus-host disease. The response rates were slightly lower in patients with DLBCL, FL, MM, and solid tumours. Bleeding, infection, and atrial fibrillation are the most concerning AEs of ibrutinib in 8-year follow-up data. The second-generation BTK inhibitor acalabrutinib has been approved for CLL/SLL and R/R MCL by the FDA. Zanubrutinib was awarded four honourable rights for expedited review, including Fast Track, Accelerated Approval, Breakthrough Therapies, and Priority Review in the United States, and has become the first Chinese-origin drug that won a grand slam tournament in FDA history. It has been approved for R/R MCL by the FDA. Most clinical trials are ongoing, and we are looking forward to the efficiency and toxicity data in long-term follow-up. The pharmacodynamics, pharmacokinetics, and indications of the three approved BTK inhibitors may differ. These differences may influence the dosage, efficiency, and AEs in clinical practice. Although the ORRs of single-agent ibrutinib, acalabrutinib, and zanubrutinib showed different rates, it is difficult to compare them directly because the baseline characteristic of the populations and the design of clinical trials may differ. Most patients in ibrutinib trials received three-line therapies, and the response assessment was the 2007 Cheson IWG criterion. In addition, the long-term follow-up data are limited for acalabrutinib and zanubrutinib, and they are not approved for any indications by the European Medicines Agency yet. Several head-to-head randomized trials are ongoing to determine which BTK inhibitor is the best-in-class drug.

Although BTK inhibitors have been approved by the FDA, many efforts are needed: (1) It is necessary to broaden the potential applications, especially for diseases with unmet clinical treatment. For instance, although the response rates reported for ibrutinib for DLBCL are still low, part of the population indeed responded. The exploration of some translational medicine tools, such as pharmacogenomic or humanized preclinical models, to distinguish benefit populations may be helpful for translational research. (2) Continuous therapy based on BTK inhibition might come out of age, and more study groups worldwide are focusing on time-limited treatment strategies as combination strategies, such as combination with the Bcl-2 inhibitor venetoclax. However, the strategies of combinations should be determined cautiously and rationally, especially for the combination of more than three drugs simultaneously. The problems of toxicity, costs, and efficacy should be balanced. (3) Conducting head-to-head randomized clinical trials directly comparing the efficacy and safety of different BTK inhibitors in specific populations, especially for the elderly, is necessary. The possible cumulative and long-term toxicity should be noted. (4) Drug resistance must be resolved. Inventing more novel agents with differential mechanisms or synergistically combining BTK inhibitors with other chemotherapy, antibodies, targeted agents, or immunotherapy may open the way for a cure in B-cell lymphomas. Last but not the least, we recommend that funding agencies, reviewers, and journal editors provide more opportunities for negative clinical data at conferences or in publications. More cooperation between physicians, scientists, and patient advocates may also accelerate the process of drug discovery and clinical development.

## Supplementary information

Supplementary Table 1

## References

[1] Teras LR, DeSantis CE, Cerhan JR, Morton LM, Jemal A, Flowers CR (2016). 2016 US lymphoid malignancy statistics by World Health Organization subtypes. CA Cancer J Clin.

[2] Siegel RL, Miller KD, Jemal A (2020). Cancer statistics, 2020. CA Cancer J Clin.

[3] Li N, Huang H, Wu D, Yang Z, Wang J, Wang J (2019). Changes in clinical trials of cancer drugs in mainland China over the decade 2009–18: a systematic review. Lancet Oncol.

[4] Hay M, Thomas DW, Craighead JL, Economides C, Rosenthal J (2014). Clinical development success rates for investigational drugs. Nat Biotechnol.

[5] Vetrie D, Vorechovsky I, Sideras P, Holland J, Davies A, Flinter F (1993). The gene involved in X-linked agammaglobulinaemia is a member of the src family of protein-tyrosine kinases. Nature.

[6] Hendriks RW, Yuvaraj S, Kil LP (2014). Targeting Bruton’s tyrosine kinase in B cell malignancies. Nat Rev Cancer.

[7] Burger JA, Wiestner A (2018). Targeting B cell receptor signalling in cancer: preclinical and clinical advances. Nat Rev Cancer.

[8] Rawlings DJ, Schwartz MA, Jackson SW, Meyer-Bahlburg A (2012). Integration of B cell responses through Toll-like receptors and antigen receptors. Nat Rev Immunol.

[9] Mahajan S, Ghosh S, Sudbeck EA, Zheng Y, Downs S, Hupke M (1999). Rational design and synthesis of a novel anti-leukemic agent targeting Bruton’s tyrosine kinase (BTK), LFM-A13 [alpha-cyano-beta-hydroxy-beta-methyl-N-(2, 5-dibromophenyl)propenamide]. J Biol Chem.

[10] Pan Z, Scheerens H, Li SJ, Schultz BE, Sprengeler PA, Burrill LC (2007). Discovery of selective irreversible inhibitors for Bruton’s tyrosine kinase. ChemMedChem.

[11] Honigberg LA, Smith AM, Sirisawad M, Verner E, Loury D, Chang B (2010). The Bruton tyrosine kinase inhibitor PCI-32765 blocks B-cell activation and is efficacious in models of autoimmune disease and B-cell malignancy. Proc Natl Acad Sci USA.

[12] Da Cunha-Bang C, Niemann CU (2018). Targeting Bruton’s Tyrosine Kinase Across B-Cell Malignancies. Drugs.

[13] Harrington BK, Gardner HL, Izumi R, Hamdy A, Rothbaum W, Coombes KR (2016). Preclinical Evaluation of the Novel BTK Inhibitor Acalabrutinib in Canine Models of B-Cell Non-Hodgkin Lymphoma. PLOS ONE.

[14] Syed YY (2020). Zanubrutinib: first approval. Drugs.

[15] Guo Y, Liu Y, Hu N, Yu D, Zhou C, Shi G (2019). Discovery of Zanubrutinib (BGB-3111), a novel, potent, and selective covalent inhibitor of Bruton’s tyrosine kinase. J Med Chem.

[16] Kaptein A, de Bruin G, Emmelot-van Hoek M, van de Kar B, de Jong A, Gulrajani M (2018). Potency and selectivity of BTK inhibitors in clinical development for B-Cell malignancies. B.

[17] Alsadhan A, Cheung J, Gulrajani M, Gaglione EM, Nierman P, Hamdy A (2020). Pharmacodynamic analysis of BTK inhibition in patients with chronic lymphocytic leukemia treated with acalabrutinib. Clin Cancer Res..

[18] Tam CS, Trotman J, Opat S, Burger JA, Cull G, Gottlieb D (2019). Phase 1 study of the selective BTK inhibitor zanubrutinib in B-cell malignancies and safety and efficacy evaluation in CLL. Blood.

[19] Wu J, Zhang M, Liu D (2016). Acalabrutinib (ACP-196): a selective second-generation BTK inhibitor. J Hematol Oncol.

[20] Lynch TJ, Kim ES, Eaby B, Garey J, West DP, Lacouture ME (2007). Epidermal growth factor receptor inhibitor-associated cutaneous toxicities: an evolving paradigm in clinical management. Oncologist.

[21] Rajasekaran N, Sadaram M, Hebb J, Sagiv-Barfi I, Ambulkar S, Rajapaksa A (2014). Three BTK-specific inhibitors, in contrast to ibrutinib, do not antagonize rituximab-dependent NK-cell mediated cytotoxicity. Blood.

[22] Barf T, Covey T, Izumi R, van de Kar B, Gulrajani M, van Lith B (2017). Acalabrutinib (ACP-196): a covalent Bruton tyrosine kinase inhibitor with a differentiated selectivity and in vivo potency profile. J Pharmacol Exp Ther.

[23] Flinsenberg TWH, Tromedjo CC, Hu N, Liu Y, Guo Y, Thia KYT (2020). Differential effects of BTK inhibitors ibrutinib and zanubrutinib on NK-cell effector function in patients with mantle cell lymphoma. Haematologica.

[24] Chang BY, Francesco M, De Rooij MF, Magadala P, Steggerda SM, Huang MM (2013). Egress of CD19(+)CD5(+) cells into peripheral blood following treatment with the Bruton tyrosine kinase inhibitor ibrutinib in mantle cell lymphoma patients. Blood.

[25] Advani RH, Buggy JJ, Sharman JP, Smith SM, Boyd TE, Grant B (2013). Bruton tyrosine kinase inhibitor ibrutinib (PCI-32765) has significant activity in patients with relapsed/refractory B-cell malignancies. J Clin Oncol.

[26] Wang ML, Rule S, Martin P, Goy A, Auer R, Kahl BS (2013). Targeting BTK with Ibrutinib in Relapsed or Refractory Mantle-Cell Lymphoma. New Engl J Med.

[27] Dreyling M, Jurczak W, Jerkeman M, Silva RS, Rusconi C, Trneny M (2016). Ibrutinib versus temsirolimus in patients with relapsed or refractory mantle-cell lymphoma: an international, randomised, open-label, phase 3 study. Lancet.

[28] Rule S, Dreyling M, Goy A, Hess G, Auer R, Kahl B (2017). Outcomes in 370 patients with mantle cell lymphoma treated with ibrutinib: a pooled analysis from three open-label studies. Br J Haematol.

[29] Cheah CY, Seymour JF, Wang ML (2016). Mantle cell lymphoma. J Clin Oncol.

[30] Goy A, Sinha R, Williams ME, Kalayoglu BS, Drach J, Ramchandren R (2013). Single-agent lenalidomide in patients with mantle-cell lymphoma who relapsed or progressed after or were refractory to bortezomib: phase II MCL-001 (EMERGE) study. J Clin Oncol.

[31] Wang M, Rule S, Zinzani PL, Goy A, Casasnovas O, Smith SD (2018). Acalabrutinib in relapsed or refractory mantle cell lymphoma (ACE-LY-004): a single-arm, multicentre, phase 2 trial. Lancet.

[32] Tam CS, Wang M, Simpson D, Opat S, Cull G, Munoz J (2019). Updated safety and efficacy data in the phase 1 trial of patients with mantle cell lymphoma (MCL) treated with Bruton tyrosine kinase (BTK) inhibitor zanubrutinib (BGB-3111). Hematol Oncol.

[33] Song Y, Zhou K, Zou D, Zhou J, Hu J, Yang H (2020). Treatment of patients with relapsed or refractory mantle cell lymphoma with zanubrutinib, a selective inhibitor of Bruton’s tyrosine kinase. Clin Cancer Res.

[34] Davids MS, Roberts AW, Seymour JF, Pagel JM, Kahl BS, Wierda WG (2017). Phase I first-in-human study of venetoclax in patients with relapsed or refractory non-Hodgkin lymphoma. J Clin Oncol.

[35] Zhao X, Bodo J, Sun D, Durkin L, Lin J, Smith MR (2015). Combination of ibrutinib with ABT-199: synergistic effects on proliferation inhibition and apoptosis in mantle cell lymphoma cells through perturbation of BTK, AKT and BCL2 pathways. Br J Haematol.

[36] Axelrod M, Ou Z, Brett LK, Zhang L, Lopez ER, Tamayo AT (2014). Combinatorial drug screening identifies synergistic co-targeting of Bruton’s tyrosine kinase and the proteasome in mantle cell lymphoma. Leukemia.

[37] Fry DW, Harvey PJ, Keller PR, Elliott WL, Meade M, Trachet E (2004). Specific inhibition of cyclin-dependent kinase 4/6 by PD 0332991 and associated antitumor activity in human tumor xenografts. Mol Cancer Ther.

[38] Chiron D, Di Liberto M, Martin P, Huang X, Sharman J, Blecua P (2014). Cell-cycle reprogramming for PI3K inhibition overrides a relapse-specific C481S BTK mutation revealed by longitudinal functional genomics in mantle cell lymphoma. Cancer Discov.

[39] Martin P, Bartlett NL, Blum KA, Park S, Maddocks K, Ruan J (2019). A phase 1 trial of ibrutinib plus palbociclib in previously treated mantle cell lymphoma. Blood.

[40] Maddocks K, Christian B, Jaglowski S, Flynn J, Jones JA, Porcu P (2015). A phase 1/1b study of rituximab, bendamustine, and ibrutinib in patients with untreated and relapsed/refractory non-Hodgkin lymphoma. Blood.

[41] Jain P, Romaguera J, Srour SA, Lee HJ, Hagemeister F, Westin J (2018). Four-year follow-up of a single arm, phase II clinical trial of ibrutinib with rituximab (IR) in patients with relapsed/refractory mantle cell lymphoma (MCL). Br J Haematol.

[42] Wang MLP, Lee HM, Chuang HM, Wagner-Bartak NM, Hagemeister FP, Westin JM (2016). Ibrutinib in combination with rituximab in relapsed or refractory mantle cell lymphoma: a single-centre, open-label, phase 2 trial. Lancet Oncol.

[43] Jerkeman M, Eskelund CW, Hutchings M, Räty R, Wader KF, Laurell A (2018). Ibrutinib, lenalidomide, and rituximab in relapsed or refractory mantle cell lymphoma (PHILEMON): a multicentre, open-label, single-arm, phase 2 trial. Lancet Haematol.

[44] Flinn IW, van der Jagt R, Kahl BS, Wood P, Hawkins TE, Macdonald D (2014). Randomized trial of bendamustine-rituximab or R-CHOP/R-CVP in first-line treatment of indolent NHL or MCL: the BRIGHT study. Blood.

[45] Wang M, Fayad L, Wagner-Bartak N, Zhang L, Hagemeister F, Neelapu SS (2012). Lenalidomide in combination with rituximab for patients with relapsed or refractory mantle-cell lymphoma: a phase 1/2 clinical trial. Lancet Oncol.

[46] Wang ML, Jain P, Lee HJ, Hagemeister FB, Samaniego F, Westin JR (2019). Frontline treatment with ibrutinib plus rituximab (IR) followed by short course R-hypercvad/MTX is extremely potent and safe in patients (age ≤ 65 years) with mantle cell lymphoma (MCL) - results of phase-II window-1 clinical trial. Blood.

[47] Jain P, Lee HJ, Steiner RE, Hagemeister FB, Samaniego F, Westin JR (2019). Frontline treatment with ibrutinib with rituximab (IR) combination is highly effective in elderly (≥65 years) patients with mantle cell lymphoma (MCL) - results from a phase II trial. Blood.

[48] Abrams ST, Lakum T, Lin K, Jones GM, Treweeke AT, Farahani M (2007). B-cell receptor signaling in chronic lymphocytic leukemia cells is regulated by overexpressed active protein kinase CbetaII. Blood.

[49] Kil LP, de Bruijn MJ, van Hulst JA, Langerak AW, Yuvaraj S, Hendriks RW (2013). Bruton’s tyrosine kinase mediated signaling enhances leukemogenesis in a mouse model for chronic lymphocytic leukemia. Am J Blood Res.

[50] Niemann CU, Herman SE, Maric I, Gomez-Rodriguez J, Biancotto A, Chang BY (2016). Disruption of in vivo chronic lymphocytic leukemia tumor-microenvironment interactions by ibrutinib–findings from an investigator-initiated phase II study. Clin Cancer Res.

[51] Zou YX, Zhu HY, Li XT, Xia Y, Miao KR, Zhao SS (2019). The impacts of zanubrutinib on immune cells in patients with chronic lymphocytic leukemia/small lymphocytic lymphoma. Hematol Oncol.

[52] Shanafelt TD, Wang XV, Kay NE, Hanson CA, O Brien S, Barrientos J (2019). Ibrutinib-rituximab or chemoimmunotherapy for chronic lymphocytic leukemia. N Engl J Med.

[53] O’Brien S, Furman RR, Coutre S, Flinn IW, Burger JA, Blum K (2018). Single-agent ibrutinib in treatment-naive and relapsed/refractory chronic lymphocytic leukemia: a 5-year experience. Blood.

[54] Byrd JC, Furman RR, Coutre SE, Flinn IW, Burger JA, Blum KA (2013). Targeting BTK with ibrutinib in relapsed chronic lymphocytic leukemia. N. Engl J Med.

[55] Munir T, Brown JR, O’Brien S, Barrientos JC, Barr PM, Reddy NM (2019). Final analysis from RESONATE: Up to six years of follow‐up on ibrutinib in patients with previously treated chronic lymphocytic leukemia or small lymphocytic lymphoma. Am J Hematol.

[56] Jain P, Keating MJ, Wierda WG, Sivina M, Thompson PA, Ferrajoli A (2017). Long-term follow-up of treatment with ibrutinib and rituximab in patients with high-risk chronic lymphocytic leukemia. Clin Cancer Res.

[57] Jaglowski SM, Jones JA, Nagar V, Flynn JM, Andritsos LA, Maddocks KJ (2015). Safety and activity of BTK inhibitor ibrutinib combined with ofatumumab in chronic lymphocytic leukemia: a phase 1b/2 study. Blood.

[58] Hillmen P, Rawstron AC, Brock K, Munoz-Vicente S, Yates FJ, Bishop R (2019). Ibrutinib plus venetoclax in relapsed/refractory chronic lymphocytic leukemia: the CLARITY study. J Clin Oncol.

[59] Byrd JC, Hillmen P, O Brien S, Barrientos JC, Reddy NM, Coutre S (2019). Long-term follow-up of the RESONATE phase 3 trial of ibrutinib vs ofatumumab. Blood.

[60] Kohrt HE, Sagiv-Barfi I, Rafiq S, Herman SE, Butchar JP, Cheney C (2014). Ibrutinib antagonizes rituximab-dependent NK cell-mediated cytotoxicity. Blood.

[61] Chanan-Khan A, Cramer P, Demirkan F, Fraser G, Silva RS, Grosicki S (2016). Ibrutinib combined with bendamustine and rituximab compared with placebo, bendamustine, and rituximab for previously treated chronic lymphocytic leukaemia or small lymphocytic lymphoma (HELIOS): a randomised, double-blind, phase 3 study. Lancet Oncol.

[62] Brown JR, Barrientos JC, Barr PM, Flinn IW, Burger JA, Tran A (2015). The Bruton tyrosine kinase inhibitor ibrutinib with chemoimmunotherapy in patients with chronic lymphocytic leukemia. Blood.

[63] Fraser G, Cramer P, Demirkan F, Silva RS, Grosicki S, Pristupa A (2019). Updated results from the phase 3 HELIOS study of ibrutinib, bendamustine, and rituximab in relapsed chronic lymphocytic leukemia/small lymphocytic lymphoma. Leukemia.

[64] Coutré SE, Furman RR, Flinn IW, Burger JA, Blum K, Sharman J (2017). Extended treatment with single-agent ibrutinib at the 420 mg dose leads to durable responses in chronic lymphocytic leukemia/small lymphocytic lymphoma. Clin Cancer Res.

[65] O’Brien S, Furman RR, Coutre SE, Sharman JP, Burger JA, Blum KA (2014). Ibrutinib as initial therapy for elderly patients with chronic lymphocytic leukaemia or small lymphocytic lymphoma: an open-label, multicentre, phase 1b/2 trial. Lancet Oncol.

[66] Burger JA, Tedeschi A, Barr PM, Robak T, Owen C, Ghia P (2015). Ibrutinib as initial therapy for patients with chronic lymphocytic leukemia. N Engl J Med.

[67] Woyach JA, Smucker K, Smith LL, Lozanski A, Zhong Y, Ruppert AS (2014). Prolonged lymphocytosis during ibrutinib therapy is associated with distinct molecular characteristics and does not indicate a suboptimal response to therapy. Blood.

[68] Burger JA, Barr PM, Robak T, Owen C, Ghia P, Tedeschi A (2020). Long-term efficacy and safety of first-line ibrutinib treatment for patients with CLL/SLL: 5 years of follow-up from the phase 3 RESONATE-2 study. Leukemia.

[69] Goede V, Fischer K, Busch R, Engelke A, Eichhorst B, Wendtner CM (2014). Obinutuzumab plus chlorambucil in patients with CLL and coexisting conditions. N Engl J Med.

[70] Woyach JA, Ruppert AS, Heerema NA, Zhao W, Booth AM, Ding W (2018). Ibrutinib Regimens versus Chemoimmunotherapy in Older Patients with Untreated CLL. N Engl J Med.

[71] O Brien SM, Jaglowski S, Byrd JC, Bannerji R, Blum KA, Fox CP (2018). Prognostic factors for complete response to ibrutinib in patients with chronic lymphocytic leukemia. JAMA Oncol.

[72] Ahn IE, Farooqui MZH, Tian X, Valdez J, Sun C, Soto S (2018). Depth and durability of response to ibrutinib in CLL: 5-year follow-up of a phase 2 study. Blood.

[73] O’Brien S, Jones JA, Coutre SE, Mato AR, Hillmen P, Tam C (2016). Ibrutinib for patients with relapsed or refractory chronic lymphocytic leukaemia with 17p deletion (RESONATE-17): a phase 2, open-label, multicentre study. Lancet Oncol.

[74] Farooqui MZH, Valdez J, Martyr S, Aue G, Saba N, Niemann CU (2015). Ibrutinib for previously untreated and relapsed or refractory chronic lymphocytic leukaemia with TP53 aberrations: a phase 2, single-arm trial. Lancet Oncol.

[75] Davids MS, Brander DM, Kim HT, Tyekucheva S, Bsat J, Savell A (2019). Ibrutinib plus fludarabine, cyclophosphamide, and rituximab as initial treatment for younger patients with chronic lymphocytic leukaemia: a single-arm, multicentre, phase 2 trial. Lancet Haematol.

[76] Moreno C, Greil R, Demirkan F, Tedeschi A, Anz B, Larratt L (2019). Ibrutinib plus obinutuzumab versus chlorambucil plus obinutuzumab in first-line treatment of chronic lymphocytic leukaemia (iLLUMINATE): a multicentre, randomised, open-label, phase 3 trial. Lancet Oncol.

[77] Byrd JC, Harrington B, O Brien S, Jones JA, Schuh A, Devereux S (2016). Acalabrutinib (ACP-196) in relapsed chronic lymphocytic leukemia. N Engl J Med.

[78] Byrd JC (2020). Acalabrutinib monotherapy in patients with relapsed/refractory chronic lymphocytic leukemia: updated phase 2 results. Blood.

[79] Awan FT, Schuh A, Brown JR, Furman RR, Pagel JM, Hillmen P (2019). Acalabrutinib monotherapy in patients with chronic lymphocytic leukemia who are intolerant to ibrutinib. Blood Adv.

[80] Woyach JA, Blachly JS, Rogers KA, Bhat SA, Jianfar M, Lozanski G (2020). Acalabrutinib plus obinutuzumab in treatment-naïve and relapsed/refractory chronic lymphocytic leukemia. Cancer Discov.

[81] Sharman JP, Egyed M, Jurczak W, Skarbnik A, Pagel JM, Flinn IW (2020). Acalabrutinib with or without obinutuzumab versus chlorambucil and obinutuzmab for treatment-naive chronic lymphocytic leukaemia (ELEVATE TN): a randomised, controlled, phase 3 trial. Lancet.

[82] Ghia P, Pluta A, Wach M, Lysak D, Kozak T, Simkovic M, et al. ASCEND: Phase III, randomized trial of acalabrutinib versus idelalisib plus rituximab or bendamustine plus rituximab in relapsed or refractory chronic lymphocytic leukemia. J Clin Oncol. 2020:O1903355. 10.1200/JCO.19.03355 32459600

[83] Xu W, Yang S, Zhou K, Pan L, Li Z, Zhou J (2019). Zanubrutinib for patients with relapsed or refractory chronic lymphocytic leukemia. Hematol Oncol.

[84] Leblond V, Kastritis E, Advani R, Ansell SM, Buske C, Castillo JJ (2016). Treatment recommendations from the Eighth International Workshop on Waldenstrom’s Macroglobulinemia. Blood.

[85] Hunter ZR, Xu L, Yang G, Zhou Y, Liu X, Cao Y (2014). The genomic landscape of Waldenstrom macroglobulinemia is characterized by highly recurring MYD88 and WHIM-like CXCR4 mutations, and small somatic deletions associated with B-cell lymphomagenesis. Blood.

[86] Yang G, Zhou Y, Liu X, Xu L, Cao Y, Manning RJ (2013). A mutation in MYD88 (L265P) supports the survival of lymphoplasmacytic cells by activation of Bruton tyrosine kinase in Waldenstrom macroglobulinemia. Blood.

[87] Treon SP, Tripsas CK, Meid K, Warren D, Varma G, Green R (2015). Ibrutinib in previously treated Waldenstrom’s macroglobulinemia. N Engl J Med.

[88] Treon SP, Gustine J, Meid K, Yang G, Xu L, Liu X (2018). Ibrutinib Monotherapy in Symptomatic, Treatment-Naïve Patients With Waldenström Macroglobulinemia. J Clin Oncol.

[89] Owen RG, McCarthy H, Rule S, D’Sa S, Thomas SK, Tournilhac O (2020). Acalabrutinib monotherapy in patients with Waldenström macroglobulinemia: a single-arm, multicentre, phase 2 study. Lancet Haematol.

[90] Noy A, de Vos S, Thieblemont C, Martin P, Flowers CR, Morschhauser F (2017). Targeting Bruton tyrosine kinase with ibrutinib in relapsed/refractory marginal zone lymphoma. Blood.

[91] Goy A, Ramchandren R, Ghosh N, Munoz J, Morgan DS, Dang NH (2019). Ibrutinib plus lenalidomide and rituximab has promising activity in relapsed/refractory non-germinal center B-cell-like DLBCL. Blood.

[92] Dasmahapatra G, Patel H, Dent P, Fisher RI, Friedberg J, Grant S (2013). The Bruton tyrosine kinase (BTK) inhibitor PCI-32765 synergistically increases proteasome inhibitor activity in diffuse large-B cell lymphoma (DLBCL) and mantle cell lymphoma (MCL) cells sensitive or resistant to bortezomib. Br J Haematol.

[93] Sagiv-Barfi I, Kohrt HEK, Czerwinski DK, Ng PP, Chang BY, Levy R (2015). Therapeutic antitumor immunity by checkpoint blockade is enhanced by ibrutinib, an inhibitor of both BTK and ITK. Proc Natl Acad Sci USA.

[94] Wilson WH, Young RM, Schmitz R, Yang Y, Pittaluga S, Wright G (2015). Targeting B cell receptor signaling with ibrutinib in diffuse large B cell lymphoma. Nat Med.

[95] Sauter CS, Matasar MJ, Schoder H, Devlin SM, Drullinsky P, Gerecitano J (2018). A phase 1 study of ibrutinib in combination with R-ICE in patients with relapsed or primary refractory DLBCL. Blood.

[96] Younes A, Sehn LH, Johnson P, Zinzani PL, Hong X, Zhu J (2019). Randomized phase III trial of ibrutinib and rituximab plus cyclophosphamide, doxorubicin, vincristine, and prednisone in non-germinal center B-cell diffuse large b-cell lymphoma. J Clin Oncol.

[97] Soussain C, Choquet S, Blonski M, Leclercq D, Houillier C, Rezai K (2019). Ibrutinib monotherapy for relapse or refractory primary CNS lymphoma and primary vitreoretinal lymphoma: Final analysis of the phase II ‘proof-of-concept’ iLOC study by the Lymphoma study association (LYSA) and the French oculo-cerebral lymphoma (LOC) network. Eur J Cancer.

[98] Bartlett NL, Costello BA, LaPlant BR, Ansell SM, Kuruvilla JG, Reeder CB (2018). Single-agent ibrutinib in relapsed or refractory follicular lymphoma: a phase 2 consortium trial. Blood.

[99] Gopal AK, Schuster SJ, Fowler NH, Trotman J, Hess G, Hou JZ (2018). Ibrutinib as treatment for patients with relapsed/refractory follicular lymphoma: results from the open-label, multicenter, phase II DAWN study. J Clin Oncol.

[100] Fowler NH, Davis RE, Rawal S, Nastoupil L, Hagemeister FB, McLaughlin P (2014). Safety and activity of lenalidomide and rituximab in untreated indolent lymphoma: an open-label, phase 2 trial. Lancet Oncol.

[101] Tai YT, Chang BY, Kong SY, Fulciniti M, Yang G, Calle Y (2012). Bruton tyrosine kinase inhibition is a novel therapeutic strategy targeting tumor in the bone marrow microenvironment in multiple myeloma. Blood.

[102] Chari A, Larson S, Holkova B, Cornell RF, Gasparetto C, Karanes C (2018). Phase 1 trial of ibrutinib and carfilzomib combination therapy for relapsed or relapsed and refractory multiple myeloma. Leukemia Lymphoma.

[103] Richardson PG, Bensinger WI, Huff CA, Costello CL, Lendvai N, Berdeja JG (2018). Ibrutinib alone or with dexamethasone for relapsed or relapsed and refractory multiple myeloma: phase 2 trial results. Br J Haematol.

[104] Younes A, Brody J, Carpio C, Lopez-Guillermo A, Ben-Yehuda D, Ferhanoglu B (2019). Safety and activity of ibrutinib in combination with nivolumab in patients with relapsed non-Hodgkin lymphoma or chronic lymphocytic leukaemia: a phase 1/2a study. Lancet Haematol.

[105] Tam CS, Cull G, Opat S, Gregory GP, Liu A, Johnston AM (2019). An update on safety and preliminary efficacy of highly specific Bruton tyrosine kinase (BTK) inhibitor zanubrutinib in combination with PD-1 inhibitor tislelizumab in patients with previously treated B-cell lymphoid malignancies. Blood.

[106] Overman M, Javle M, Davis RE, Vats P, Kumar-Sinha C, Xiao L (2020). Randomized phase II study of the Bruton tyrosine kinase inhibitor acalabrutinib, alone or with pembrolizumab in patients with advanced pancreatic cancer. J Immunother Cancer.

[107] Al-Toubah T (2020). A Phase II Study of Ibrutinib in Advanced Neuroendocrine Neoplasms. Neuroendocrinology.

[108] Hong D, Rasco D, Veeder M, Luke JJ, Chandler J, Balmanoukian A (2019). A phase 1b/2 study of the Bruton tyrosine kinase inhibitor ibrutinib and the PD-L1 inhibitor durvalumab in patients with pretreated solid tumors. Oncology.

[109] Coutre SE, Byrd JC, Hillmen P, Barrientos JC, Barr PM, Devereux S (2019). Long-term safety of single-agent ibrutinib in patients with chronic lymphocytic leukemia in 3 pivotal studies. Blood Adv.

[110] Woyach JA, Furman RR, Liu TM, Ozer HG, Zapatka M, Ruppert AS (2014). Resistance mechanisms for the Bruton’s tyrosine kinase inhibitor ibrutinib. N Engl J Med.

[111] Zhang, L, et al., Metabolic reprogramming toward oxidative phosphorylation identifies a therapeutic target for mantle cell lymphoma. Sci Transl Med, 2019;11. 10.1126/scitranslmed.aau1167 31068440

[112] Xu L, Tsakmaklis N, Yang G, Chen JG, Liu X, Demos M (2017). Acquired mutations associated with ibrutinib resistance in Waldenstrom macroglobulinemia. Blood.

[113] Cull G (2019). Treatment with the Bruton Tyrosine Kinase Inhibitor Zanubrutinib (BGB-3111) Demonstrates High Overall Response Rate and Durable Responses in Patients with Chronic Lymphocytic Leukemia/Small Lymphocytic Lymphoma (CLL/SLL): Updated Results from a Phase 1/2 Trial. Blood.

[114] Sun C (2020). Clinical and biological implications of target occupancy in CLL treated with the BTK inhibitor acalabrutinib. Blood.

[115] Rule S (2018). Ibrutinib versus temsirolimus: 3-year follow-up of patients with previously treated mantle cell lymphoma from the phase 3, international, randomized, open-label RAY study. Leukemia.

[116] Tam CS (2018). Ibrutinib plus Venetoclax for the Treatment of Mantle-Cell Lymphoma. The New England Journal of Medicine.

[117] Dimopoulos MA (2018). Phase 3 Trial of Ibrutinib plus Rituximab in Waldenstrom’s Macroglobulinemia. N Engl J Med.

[118] Trotman J, Opat S, Marlton P (2019). Updated safety and efficacy data in a phase 1/2 trial of patients with Waldenstrom macroglobulinaemia (WM) treated with the Bruton tyrosine kinase (BTK) inhibitor zanubrutinib (BGB-3111) [abstract no. PF481]. HemaSphere..

[119] Dimopoulos M, Opat S, Lee HP (2019). Major responses in Myd88 wildtype (Myd88wt) Waldenstrom macroglobulinemia (WM) patients treated with Bruton tyrosine kinase (BTK) inhibitor zanubrutinib (BGB-3111) [abstract no. PF487]. HemaSphere..

[120] Tam, CS, et al., A randomized phase 3 trial of zanubrutinib versus ibrutinib in symptomatic waldenstrom macroglobulinemia:the aspen study. Blood, 2020. 10.1182/blood.2020006844 PMC7596850 32731259

[121] Younes AP (2014). Combination of ibrutinib with rituximab, cyclophosphamide, doxorubicin, vincristine, and prednisone (R-CHOP) for treatment-naïve patients with CD20-positive B-cell non-Hodgkin lymphoma: a non-randomised, phase 1b study. Lancet Oncology, The.

[122] Grommes C (2017). Ibrutinib Unmasks Critical Role of Bruton Tyrosine Kinase in Primary CNS Lymphoma. Cancer Discov.

[123] Bartlett NL (2014). Ibrutinib Monotherapy in Relapsed/Refractory Follicular Lymphoma (FL): Preliminary Results of a Phase 2 Consortium (P2C) Trial. Blood.

[124] Ujjani CS (2016). Phase 1 trial of rituximab, lenalidomide, and ibrutinib in previously untreated follicular lymphoma: Alliance A051103. Blood.

